# Inequalities in access to food in Brazil: a scoping review with a racial and gender focus and policy recommendations

**DOI:** 10.1590/0102-311XEN211325

**Published:** 2026-07-06

**Authors:** Letícia Lopes Vieira, Raquel Canuto, Ana Maria Thomaz Maya Martins, Laís Amaral Mais, Larissa Loures Mendes, Luana Lara Rocha

**Affiliations:** 1 Universidade Federal de Minas Gerais, Belo Horizonte, Brasil.; 2 Universidade Federal do Rio Grande do Sul, Porto Alegre, Brasil.; 3 Instituto de Defesa de Consumidores, São Paulo, Brasil.

**Keywords:** Food Security, Gender, Racial Groups, Public Policy, Segurança Alimentar, Gênero, Grupos Raciais, Política Pública, Seguridad Alimentaria, Género, Grupos Raciales, Política Pública

## Abstract

Vulnerable populations, such as women and Black individuals, face greater physical and economic challenges in accessing food, revealing that food environments are structured as a reflection of the country’s social inequalities. In this context, this study aimed to conduct a scoping review in order to identify studies on food access, acquisition, and availability in Brazil, with a specific focus on gender and race/skin color. The study also aimed to identify recommendations for policymakers in designing public policies to improve food access in Brazil, while considering the existing inequalities across these dimensions. The search was conducted in four databases without date restrictions. The search strategy was constructed by combining the descriptors “access”, “acquisition”, and “availability” of food with social markers such as “gender” and “race/skin color”, using Boolean operators. All stages of study selection and data extraction were conducted by two independent researchers. The findings reveal structural inequalities in food access, with more pronounced barriers for Black women. A concentration of food deserts and swamps was observed in areas with greater social and racial vulnerability. Nonetheless, a lack of studies addressing gender and the intersection of gender and race/skin color was identified. The results of this review underscore the urgency of fair public policies that incorporate racial and gender equity and can ensure the human right to adequate food equitably for the entire Brazilian population.

## Abstract (pt)

Populações em situações de vulnerabilidade, como mulheres e pessoas negras, enfrentam maiores desafios físicos e econômicos no acesso à alimentação, evidenciando que os ambientes alimentares são estruturados como um reflexo das desigualdades sociais do país. Nesse contexto, o objetivo deste estudo foi realizar uma revisão de escopo para identificar literatura sobre acesso, aquisição e disponibilidade de alimentos no Brasil, com foco específico em gênero e raça/cor da pele. Além disso, buscou-se identificar recomendações aos gestores para a formulação de políticas públicas voltadas à melhoria do acesso à alimentação no país, considerando as desigualdades existentes nessas dimensões. A busca foi realizada em quatro bases de dados, sem restrição de data. A estratégia de busca foi construída combinando os descritores “acesso”, “aquisição” e “disponibilidade” de alimentos com marcadores sociais como “gênero” e “raça/cor da pele”, utilizando operadores booleanos. Todas as etapas de seleção de estudos e extração de dados foram realizadas de forma independente por dois pesquisadores. Os achados revelam desigualdades estruturais no acesso à alimentação, com barreiras mais pronunciadas para mulheres negras. Observou-se uma concentração de desertos e pântanos alimentares em áreas de maior vulnerabilidade social e racial. Contudo, identificou-se uma escassez de estudos abordando o gênero e a interseção entre gênero e raça/cor da pele. Os resultados desta revisão ressaltam a urgência de políticas públicas justas, que incorporem a equidade racial e de gênero e que sejam capazes de assegurar o direito humano à alimentação adequada de forma equitativa a toda a população brasileira.

## Abstract (es)

Las poblaciones en situaciones de vulnerabilidad, como las mujeres y las personas negras, se enfrentan a mayores dificultades físicas y económicas para acceder a los alimentos, lo que pone de manifiesto que los entornos alimentarios están estructurados como un reflejo de las desigualdades sociales del país. En este contexto, el objetivo de este estudio fue realizar una revisión de alcance para identificar estudios sobre el acceso, la adquisición y la disponibilidad de alimentos en Brasil, con un enfoque específico en el género y la raza/color de piel, así como identificar recomendaciones para los responsables políticos en el diseño de políticas públicas para mejorar el acceso a los alimentos en el país, teniendo en cuenta las desigualdades existentes con respecto a estas dimensiones. La búsqueda se realizó en cuatro bases de datos sin restricciones de fecha. La estrategia de búsqueda se elaboró combinando los descriptores “acceso”, “adquisición” y “disponibilidad” de alimentos con marcadores sociales como “género” y “raza/color de piel”, utilizando operadores booleanos. Todas las etapas de selección de estudios y extracción de datos fueron realizadas de forma independiente por dos investigadores. Los resultados revelan desigualdades estructurales en el acceso a los alimentos, con barreras más pronunciadas para las mujeres negras. Se observó una concentración de desiertos y pantanos alimentarios en zonas con mayor vulnerabilidad social y racial. No obstante, se identificó una falta de estudios que abordaran el género y la intersección entre género y raza/color de piel. Los resultados de esta revisión subrayan la urgencia de políticas públicas justas que incorporen la equidad racial y de género y sean capaces de garantizar el derecho humano a una alimentación adecuada de manera equitativa para toda la población brasileña.

## Introduction

In recent decades, the industrialization process has transformed the methods of food production and distribution in Brazil, influencing how the population accesses and acquires food ^1^. Data from the 2017-2018 *Brazilian Household Budget Survey* (POF, acronym in Portuguese), reveal that supermarkets and hypermarkets were the main places for food purchases in the country, while other studies indicate that traditional markets, such as farmers’ markets and small grocery stores, once central to this process, have been losing ground in this sector ^2^ ^,^ ^3^.

These transformations have reshaped food environments, understood as the physical, economic, political, and sociocultural contexts that shape how people access, acquire, store, prepare, and consume food ^4^ ^,^ ^5^, because of changes in the availability and type of establishments, marketing strategies, and population access patterns ^6^. However, the configuration of these environments is shaped by structural inequalities, marked by historical processes of discrimination and oppression that disproportionately affect ethnic-racial minority groups and women, resulting in inequities in access to adequate and healthy foods ^3^ ^,^ ^7^ ^,^ ^8^.

A systematic review aimed at assessing ethnic-racial inequities in community and consumer food environments worldwide found that Black populations are disproportionately affected by food deserts and food swamps ^9^. These groups endure lower availability of supermarkets and outlets selling healthy foods, and greater exposure to establishments selling unhealthy foods and to related advertising ^10^. Additionally, a review conducted by Castronuovo et al. ^8^ indicated that women are particularly susceptible to food and nutrition insecurity, while Pereira et al. ^10^ found that households headed by Black women were more likely to experience moderate to severe food and nutrition insecurity.

In this context, vulnerable populations, such as women and Black individuals, face greater challenges in both physical and economic access to food, demonstrating that food environments are organized as a reflection of the country’s social inequalities ^9^ ^,^ ^11^. These unfair inequalities affect multiple dimensions of the food environment as well as the possibilities for choosing and consuming healthy foods, thereby perpetuating inequities in the human right to adequate food ^12^.

Recognizing how these inequities operate is essential for planning and implementing effective and intersectoral public policies that promote health, social justice, and the fulfilment of the human right to adequate food. Accordingly, this study aims to conduct a scoping review of studies on food access, acquisition, and availability in Brazil, with a specific focus on gender and race/skin color, and to identify recommendations for decision-makers in the formulation of public policies aimed at improving access to adequate and healthy food in the country, while considering the existing inequalities across these dimensions.

This scoping review makes a distinct contribution by offering the first synthesis of evidence on food access, acquisition, and availability in Brazil by an explicit intersectional lens that considers both gender and race/skin color. This also uniquely incorporates policy-oriented institutional reports, enabling the review to map not only empirical findings but also actionable recommendations for public policy, an aspect not previously emphasized in the literature. By examining multiple dimensions of food access simultaneously, the review provides a comprehensive picture of the structural barriers shaping food inequities in the country.

## Methodology

A scoping review was conducted on food access, acquisition, and availability in Brazil, with a focus on gender and race/skin color, following the *Preferred Reporting Items for Systematic Reviews and Meta-Analyses Extension for Scoping Reviews* (PRISMA-ScR) guidelines to ensure a robust and reproducible process. Although a protocol was developed to guide this scoping review, it was not prospectively registered, as protocol registration is not a requirement under PRISMA-ScR.

### Research question

The research question was formulated based on the Population, Concept, and Context (PCC) framework proposed by the Joanna Briggs Institute ^13^: Population (P) - the Brazilian population, with data disaggregated by gender and race/skin color; Concept (C) − food access, acquisition, and availability, including barriers, inequalities, and recommendations; and Context (C) - the Brazilian territory, focusing on scientific literature and institutional reports showing data on food access, acquisition, and availability disaggregated by gender and race/skin color.

In this review, “barriers” refer to structural, economic, territorial, or social obstacles that limit individuals’ ability to access, acquire, or afford healthy foods in Brazil. “Inequalities” are understood as systematic and socially produced differences in these dimensions according to gender and race/skin color, reflecting broader patterns of discrimination and unequal distribution of opportunities. “Recommendations” are defined as policy- or program-oriented actions proposed by authors or institutional reports to reduce such inequities and improve access to adequate and healthy foods.

Based on this framework, aiming to understand gender and race/skin color inequalities in food access, acquisition, and availability in Brazil, the following research question was made: “What evidence is available on food access, acquisition, and availability in Brazil, focusing on gender and race/skin color inequalities, and what recommendations have been identified to promote food equity?”.

### Search strategy

A systematic search for published studies was conducted on May 27, 2025, in the online databases PubMed (MEDLINE), Scopus, Web of Science, and SciELO. The search strategy was structured using terms derived from Medical Subject Headings (MeSH), combining keywords such as “access”, “food”, “availability”, “price”, “acquisition”, “purchase”, “gender”, “sex”, “race”, “inequality”, and “inequity”, to identify studies and documents aligned with this review’s eligibility criteria. Supplementary Material (https://cadernos.ensp.fiocruz.br/static//arquivo/suppl-e00211325_1433.pdf) shows the descriptors used and the full search strategy for each database. The same search string was applied across PubMed, Scopus, and Web of Science to ensure consistency in the identification of studies.

A manual search was also performed in the reference lists of studies selected for full-text screening to identify potentially relevant additional works. This strategy enabled the retrieval of studies not captured in the initial electronic search.

Searches were conducted without restrictions on language or date of publication. All database searches were performed simultaneously, and references were exported to Rayyan (https://www.rayyan.ai/) for duplicate removal and study selection.

Given the nature of the research question, the search strategy was complemented by publications from national and international organizations involved in promoting adequate and healthy diets, guaranteeing human rights, and reducing gender and racial inequalities. Institutional and nongovernmental organization (NGO) websites were selected because they are recognized sources of national data and policy documents related to food and nutrition security, inequality, and human rights in Brazil. Searches on these websites were conducted manually using the same keywords applied in the database search (“access”, “availability”, “price”, “acquisition”, “purchase”, “gender”, “sex”, “race”, “inequality”, “inequity”) and by navigating menus and document repositories to identify reports that met the eligibility criteria. Institutional websites consulted included the Brazilian Institute of Geography and Statistics (IBGE; https://www.ibge.gov.br/), the Institute for Applied Economic Research (IPEA; https://www.ipea.gov.br/), the Brazilian Ministry of Health (https://www.gov.br/saude/pt-br), the Brazilian Ministry of Social Development, Family, and the Fight Against Hunger (https://www.gov.br/mds/pt-br), the Brazilian Ministry of Agrarian Development and Family Farming (https://www.gov.br/mda/pt-br), and the Food and Agriculture Organization of the United Nations (FAO; https://www.fao.org/home/en).

Moreover, websites of Brazilian third-sector organizations relevant to the topic were consulted, including the Consumer Defense Institute (IDEC; https://idec.org.br/), Alliance for Adequate and Healthy Food (https://alimentacaosaudavel.org.br/), ACT Health Promotion (https://actbr.org.br/), and Food of Tomorrow Institute (https://www.comidadoamanha.org/).

### Eligibility criteria

Original studies published online in peer-reviewed journals or institutional reports that explicitly addressed gender and/or race/skin color inequalities in food access, acquisition, or availability in Brazil were included. Studies were included when they had results stratified by gender and/or race/skin color, regardless of whether inequalities were explicitly framed as the primary analytical focus. Studies with different methodological designs were eligible. In addition to peer-reviewed articles, institutional reports, and book chapters published by recognized governmental or nongovernmental organizations were also eligible for inclusion. Studies focusing exclusively on food consumption without directly addressing access, acquisition, or availability in relation to gender and/or race/skin color inequalities were excluded. Literature reviews (narrative, systematic, or scoping) and conference abstracts without full-text articles were also excluded.

### Study selection

**Figure 1:**
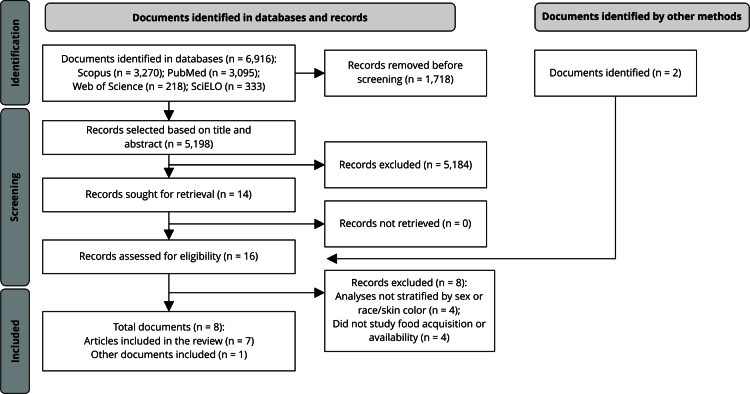
Flow diagram of the article selection process for the review.

Two reviewers independently selected the article using the Rayyan platform. In the first stage, titles and abstracts were screened for eligibility. In the second stage, selected articles underwent full-text review. Any disagreements between reviewers were resolved by a third reviewer during both the abstract screening and full-text review stages. Figure 1 summarizes the study selection process.

### Data extraction

Extracted variables included: journal and year of publication, publication language, region of Brazil studied, geographic scope (capital, metropolitan area, rural/interior), study population, study objective, database used (primary or secondary), study design, type of statistical analysis, method of data stratification by gender and race/skin color, type of foods examined (unprocessed, minimally processed, culinary ingredients, processed, or ultra-processed), main findings on food access and/or acquisition (related to gender and race/skin color), recommendations, best practices, barriers and challenges identified by the authors, and study limitations. Data extraction was conducted independently by two reviewers using a standardized extraction form developed in Google Forms (https://docs.google.com/forms) for this purpose. Any discrepancies in data extraction were resolved by a third reviewer.

### Data synthesis

Data synthesis involved identifying, categorizing, and analyzing information related to food access, acquisition, and availability in Brazil, emphasizing differences by gender and race/skin color. Additionally, the synthesis sought to identify recommendations for decision-makers on how to mitigate and reduce access inequalities via public policies. Extracted information formed a narrative synthesis of the findings. The categories used in the synthesis were developed inductively from the extracted data, rather than defined a priori, enabling themes to emerge directly from the evidence. Since this review aimed to provide an overview of food access, acquisition, and availability in Brazil by gender and race/skin color, study quality was not assessed ^14^.

## Results

The search strategy retrieved 6,916 records from electronic databases. After duplicate removal (n = 1,718), 5,198 unique records were screened, and 14 articles identified via database searches were assessed for full-text eligibility. Two full texts were also assessed from other sources: one institutional report identified via institutional website searches and one article identified via reference list screening. Of the 16 full texts assessed, seven peer-reviewed articles and one institutional report met the inclusion criteria and were included (Figure 1). The complete list of included articles, along with a description of their main characteristics, is provided in Box 1.

**Box 1:** Description of articles included in the scoping review on food access, acquisition, and availability in Brazil, with a specific focus on racial and gender.

STUDY (YEAR)	JOURNAL/PUBLISHER	LANGUAGE	REGION OF BRAZIL	GEOGRAPHIC SCOPE	STUDY POPULATION	STUDY OBJECTIVE	DATABASE USED	STUDY DESIGN	STRATIFIED BY	FOOD TYPE STUDIED
Bezerra & Sichieri ^20^ (2010)	*Revista de Saúde Pública*	English and Portuguese	Nationwide (Brazil)	All regions	Adolescents, adults, and older adults	To verify whether the diversity of food acquisition and regional socioeconomic conditions are related to the spatial distribution of overweight and obesity in the Brazilian population	POF 2002/2003	Cross-sectional	Sex	All foods
Borges et al. ^16^ (2024)	*Food Security*	English	South	Capital	Not applicable	To describe the presence of food deserts in a capital city in southern Brazil and their relationship with socioeconomic characteristics	Food retail establishment database (secondary data)	Ecological	Race/Skin color	Unprocessed, minimally processed, and ultra-processed foods
Borges et al. ^15^ (2025)	*Ciência & Saúde Coletiva*	English	South	Capital	Not applicable	To describe accessibility to farmers’ markets in Porto Alegre, Rio Grande do Sul, using four modes of transport (walking, bicycle, bus, and car) and its association with individuals’ income and race/skin color	2010 *Demographic Census* (sociodemographic data) and food retail establishment database (secondary data)	Ecological	Race/Skin color	Unprocessed, minimally processed, and ultra-processed foods
Honório et al. ^17^ (2021)	*International Journal for Equity in Health*	English	Southeast	Capital	Not applicable	To identify and compare neighborhoods classified as food deserts and food swamps based on social inequality	2010 *Demographic Census* (sociodemographic data) and food retail establishment database (secondary data)	Ecological	Race/Skin color	Unprocessed, minimally processed, and ultra-processed foods
Serafim et al. ^18^ (2022)	*Frontiers in Nutrition*	English	Southeast	Countryside (medium-sized municipality)	Not applicable	To assess the availability of ultra-processed foods according to different types of food retail establishments and sociodemographic factors in a medium-sized Brazilian municipality	Primary data collection on food establishments (AUDITNOVA)	Ecological	Race/Skin color	Ultra-processed foods
Sgambato et al. ^21^ (2022)	*Ciência & Saúde Coletiva*	English	Nationwide	All regions	Adults	To assess food expenditures according to the different social profiles of household heads in Brazil	POF 2017/2018	Cross-sectional	Race/Skin color; sex	All foods
Silva et al. ^19^ (2024)	*Journal of Biosocial Science*	English	Northeast	Capital	Not applicable	To identify food deserts and food swamps and examine their association with the socioeconomic and demographic conditions of census tracts in a northeastern Brazilian metropolis	Food retail establishment database (secondary data)	Ecological	Race/Skin color	Unprocessed, minimally processed, and ultra-processed foods
Passos et al. ^28^ (2024)	IPEA	Portuguese	Nationwide	All regions	Adults	To investigate the consumption patterns of Brazilian households, with an emphasis on the gender and race of the household head (or reference person)	POF 2017/2018	Cross-sectional	Race/Skin color; sex	All foods

IPEA: Institute for Applied Economic Research; POF: *Brazilian Household Budget Survey*.

The studies included in this review were published from 2010 to 2025, with a concentration in 2024 (37.5%; n = 3) and 2022 (25%; n = 2). All articles (n = 7) were published in English, one of which also had a Portuguese version. The report included in this review (n = 1) was published in Portuguese. Regarding geographic distribution, 37.5% of the studies (n = 3) covered all regions of Brazil. Notably, no study focused on the North or Center-West regions. In terms of spatial scope, 50% of the studies analyzed Brazilian capitals (n = 4), and 37.5% included municipalities from the countryside, capitals, and metropolitan areas simultaneously (n = 3). Ecological studies predominated (62.5%; n = 5), followed by cross-sectional studies (37.5%; n = 3). Most investigations used secondary data (87.5%; n = 7), and only one study employed primary data. Table 1 describes this information.

**Table 1:** General characteristics of the documents included in the review (n = 8).

Characteristics	n (%)
Publication year	
2010	1 (12.5)
2021	1 (12.5)
2022	2 (25.0)
2024	3 (37.5)
2025	1 (12.5)
Language of publication	
English	6 (75.0)
Portuguese	1 (12.5)
English and Portuguese	1 (12.5)
Region of Brazil	
All regions	3 (37.5)
North	0 (0.0)
Northeast	1 (12.5)
South	2 (25.0)
Southeast	2 (25.0)
Central-West	0 (0.0)
Geographic scope	
All regions	3 (37.5)
Capital	4 (50.0)
Metropolitan area	0 (0.0)
Countryside	1 (12.5)
Study design	
Ecological	5 (62.5)
Cross-sectional	3 (37.5)
Data source	
Primary	1 (12.5)
Secondary	7 (87.5)

The main object of the studies included in this review was the availability of food outlets, which was the focus of four articles (Box 2). The most frequently conducted stratified analysis was by race/skin color (n = 7), with one document also considering the intersectionality between gender and race/skin color. The most common comparison was between White individuals and other groups (Black, Mixed-race, Yellow, or Indigenous), with particular emphasis on Black and Brown categories. Regarding the types of foods analyzed, unprocessed, minimally processed, and ultra-processed foods were the most frequent in studies on food outlet availability (n = 4). In contrast, analyses encompassing all food types were more common in studies addressing food acquisition (n = 2) and food expenditure (n = 1).

**Box 2:** Characteristics of analyses on food access, acquisition, or availability in Brazil and forms of stratification for examining differences by gender or race/skin color.

STRATIFICATION OF ANALYSIS	CHARACTERISTICS OF THE STRATIFICATION VARIABLE	VARIABLE UNDER ANALYSIS	TYPE OF FOODS STUDIED	STUDY (YEAR)
Gender	Female, male	Food acquisition	All foods	Bezerra & Sichieri ^20^ (2010)
Race/Skin color	White, Black/Mixed-race, or ethnic-racial minorities (Black, Mixed-race, Yellow, and Indigenous)	Availability of food retail establishments	Unprocessed, minimally processed, and ultra-processed foods	Borges et al. ^16^ (2024); Borges et al. ^15^ (2025); Honório et al. ^17^ (2021); Silva et al. ^19^ (2024)
Race/Skin color	White, other races/skin colors	Food availability	Ultra-processed foods	Serafim et al. ^18^ (2022)
Race/Skin color	White, Black/Mixed-race	Food acquisition	All foods	Sgambato et al. ^21^ (2022)
Gender and race/skin color	White, Black; man, woman; White man, Black man, White woman, Black woman	Food expenditure	All foods	Passos et al. ^28^ (2024)

Analysis of the included studies revealed disparities in food access, acquisition, and availability in Brazil when stratified by gender and race/skin color (Box 3). Concerning gender, men showed a higher frequency of purchasing soft drinks, ready-to-eat meals, fast food, and alcoholic beverages, while women purchased more fruits, sweets, and cookies. When considering the intersection between gender and race, White women with higher education levels purchased more vegetables, fruits, and dairy products than White men, whereas Black or Mixed-race women with lower education levels spent less on soft drinks, alcoholic beverages, and ready-to-eat foods.

**Box 3:** Systematization of the main results on food access, acquisition, or availability in Brazil according to gender or race/skin color.

STRATIFICATION OF ANALYSIS	MAIN RESULTS	DATA COLLECTION PERIOD	STUDY (YEAR)
Male	Higher frequency of purchasing soft drinks, milk and dairy products, ready-to-eat meals, fast food, fried and baked snacks, and alcoholic beverages among men	2002/2003	Bezerra & Sichieri ^20^ (2010)
Female	Higher frequency of purchasing cookies, fruits, and sweets among women	2002/2003	Bezerra & Sichieri ^20^ (2010)
Black, Mixed-race, or Indigenous	Higher frequency of food deserts in regions with a high concentration of Black, Mixed-race, or Indigenous people	2020; 2019	Borges et al. ^16^ (2024); Silva et al. ^19^ (2024)
Black, Mixed-race, Indigenous, or Yellow	Lower frequency of establishments selling unprocessed and minimally processed foods in regions with a high concentration of racial minorities; none identified in areas characterized by both a high concentration of racial minorities and low income	2020/2022	Borges et al. ^15^ (2025)
Black, Mixed-race, Indigenous, or Yellow	In regions with a high concentration of racial minorities, the travel time to reach establishments selling unprocessed and minimally processed food was longer across all transportation modes (walking, bicycle, bus, and car)	2020/2022	Borges et al. ^15^ (2025)
Black or Mixed-race	Higher frequency of food deserts and food swamps in regions with a high concentration of Black or Mixed-race populations	2015	Honório et al. ^17^ (2021)
Black, Mixed-race, Indigenous, or Yellow	Regions with a high concentration of Black, Mixed-race, Yellow, or Indigenous populations showed higher averages of ultra-processed food availability	2017/2018	Serafim et al. ^18^ (2022)
White female	White women with higher education levels had greater acquisition of vegetables, fruits, and cheese compared to white men with medium education	2017/2018	Sgambato et al. ^21^ (2022)
White female	White women with lower education levels had greater acquisition of vegetables, legumes, and tubers compared to white men with medium education	2017/2018	Sgambato et al. ^21^ (2022)
Black or Mixed-race female	Black or Mixed-race women with lower education levels had lower expenditures on soft drinks, alcoholic beverages, and ready-to-eat foods	2017/2018	Sgambato et al. ^21^ (2022)
Black, Mixed-race, or Indigenous	Food swamps showed a lower proportion of Black, Mixed-race, or Indigenous populations	2019	Silva et al. ^19^ (2024)
Black or Mixed-race	Households headed by Black or Mixed-race individuals had higher food expenditures relative to total household expenses compared with other groups	2017/2018	Passos et al. ^28^ (2024)

Regarding race/skin color, the studies reported a greater concentration of food deserts and food swamps in regions with a high proportion of Black, Mixed-race, Indigenous, and Yellow populations (n = 3). In these areas, there was a lower frequency of establishments selling unprocessed and minimally processed foods, and when such outlets were available, the travel time to access them was longer for these groups across all transportation modes. Moreover, the availability of ultra-processed foods was higher in territories with a high concentration of racial minorities, and food expenditures were greater in households headed by Black or Brown individuals.

Among the eight documents included in this review, five had public policy recommendations aimed at improving access to, acquisition of, and availability of healthy foods (Box 4). The implementation of public food and nutrition security facilities, such as street markets, was widely mentioned (n = 4), as well as the promotion of urban agriculture, particularly community gardens (n = 2).

**Box 4:** Recommendations from study authors to promote access, acquisition and availability of healthy foods.

RECOMMENDATION	STUDY (YEAR)
Implementation of public food and nutrition security facilities	Borges et al. ^16^ (2024); Honório et al. ^17^ (2021); Serafim et al. ^18^ (2022); Silva et al. ^19^ (2024)
Promotion of urban agriculture (community gardens)	Borges et al. ^16^ (2024); Sgambato et al. ^21^ (2022)
Consideration of individuals’ place of residence and availability of opportunities to access food during the development of public policies	Borges et al. ^16^ (2024)
Combating all kinds of discrimination and inequality (including those related to race, income, education, housing, and sanitation) to build healthier food environments	Borges et al. ^16^ (2024); Serafim et al. ^18^ (2022); Sgambato et al. ^21^ (2022); Silva et al. ^19^ (2024)
Identification of areas with the greatest need for policies aimed at expanding access to healthy foods	Honório et al. ^17^ (2021)
Structural actions to reduce population exposure and access to unhealthy foods (e.g., taxation of ultra-processed foods, regulation of food advertising)	Honório et al. ^17^ (2021); Serafim et al. ^18^ (2022); Sgambato et al. ^21^ (2022); Silva et al. ^19^ (2024)
Identification of the population’s social profile and how it influences food acquisition patterns	Sgambato et al. ^21^ (2022)
Reduction in the price of healthy foods	Serafim et al. ^18^ (2022); Sgambato et al. ^21^ (2022); Silva et al. ^19^ (2024)

Several authors emphasized the importance of considering the place of residence and available opportunities when formulating public policies, as well as the need for structural actions to reduce the consumption of unhealthy foods, such as taxation of ultra-processed foods and regulation of food advertising (n = 4). Additionally, lowering the prices of healthy foods was highlighted as an essential measure to expand access (n = 3).

The studies also underscored the urgency of addressing all forms of inequality and discrimination, particularly racial, economic, and territorial, as a prerequisite for equitable food environments (n = 4). Finally, the importance of identifying the most vulnerable territories and the social profiles of the population was emphasized as a key input for developing effective and targeted public policies (n = 2).

The limitations described below refer to methodological constraints reported by the authors of the included studies and are shown here as part of the mapped evidence. The main limitations of the studies included in this review were related to the use of secondary data and reliance on sociodemographic information from the 2010 *Demographic Census*, which may be outdated. Regarding secondary data on food outlets, some studies noted the possible exclusion of informal food vendors, such as street vendors. Other limitations mentioned included the inability to draw causal inferences at the individual level in ecological studies, and the classification of food deserts and swamps, which remain under development and may show methodological constraints. Moreover, studies focusing on specific municipalities or urban contexts reported limited external validity.

## Discussion

The results of this scoping review highlight the existence of gender and race/skin color inequalities in food access, acquisition, and availability in Brazil. Although based on a limited number of studies, these findings point to emerging patterns of inequality that warrant further investigation. Most identified studies focused on the availability of food outlets, showing that regions with a high proportion of Black, Mixed-race, Indigenous, and Yellow populations have fewer outlets selling unprocessed and minimally processed foods, as well as greater exposure to food deserts and food swamps ^15^ ^,^ ^16^ ^,^ ^17^ ^,^ ^18^ ^,^ ^19^. At the same time, the absence of studies focused on the North and Central-West regions, the predominance of ecological designs based on secondary data, and the limited examination of gender and race/skin color intersectionality underscore that this is an emerging field marked by important territorial, methodological, and analytical gaps.

Regarding gender inequalities, although less frequently addressed in the studies, food acquisition patterns were also found to differ between men and women. Men showed a higher frequency of purchasing unhealthy foods ^20^, while women - especially White and those with higher levels of education - purchased more fruits, vegetables, and dairy products ^21^. Among Black or Mixed-race women with lower educational levels, expenditures on sweetened beverages, alcoholic drinks, and ultra-processed foods were lower, which may reflect economic constraints rather than dietary preferences ^21^. Few studies have explored the intersectional relationships between gender and race/skin color, which limits a more comprehensive understanding of the multiple dimensions of food access inequalities in Brazil. This lack of intersectional analyses reflects a gap in the existing literature and constitutes one of the central findings of this scoping review, highlighting the need for future research that explicitly adopts intersectional approaches.

These disparities become even more critical in areas characterized by a high concentration of racial minorities and low income, in which establishments selling healthy foods are scarce or nonexistent, reinforcing the phenomenon known as food deserts ^16^ ^,^ ^17^ ^,^ ^19^. Moreover, evidence from other countries supports this pattern, showing that areas classified as food deserts tend to have poorer socioeconomic conditions: lower per capita income; lower education levels; limited access to essential services, and a higher proportion of historically marginalized populations, such as Black, Mixed-race, and Indigenous peoples ^22^ ^,^ ^23^.

In addition to the limited presence of such establishments, evidence suggests that the travel time required to access points selling healthy foods is longer across all transportation modes (walking, cycling, bus, and car), amplifying the physical and economic barriers to the human right to adequate food ^15^. Limited access to these foods, resulting from both geographic inaccessibility and low supply, reinforces territorial, social, and economic inequalities, contributing to unhealthy dietary patterns. In contexts marked by multiple vulnerabilities, these barriers constrain the fulfilment of the human right to adequate food, perpetuating a cycle of deprivation that disproportionately affects historically marginalized groups ^24^.

At the same time, the availability of ultra-processed foods is higher in territories predominantly inhabited by historically marginalized racial groups ^18^. This concentration is exacerbated by commercial strategies that intentionally target low-income regions for the distribution and marketing of ultra-processed foods, where the supply of unprocessed or minimally processed foods is scarce ^25^.

Among the limited number of studies included in this review, only one directly addressed gender inequalities in food acquisition, representing an important gap in the literature. That study found that men purchased ultra-processed foods and alcoholic beverages more frequently, while women purchased more fruits, cookies, and sweets ^20^. This pattern was also identified in studies on food consumption using data from the *Surveillance of Risk and Protective Factors for Chronic Diseases by Telephone Survey* (Vigitel, acronym in Portuguese), which found higher consumption of fatty meats among men and higher intake of fruits, vegetables, and sweets among women, indicating gender differences in eating practices that have implications for health ^26^.

Considering the intersectionality between gender and race/skin color, it was observed that White women with higher education purchased more healthy foods, such as fruits and vegetables, compared to White men ^21^. Conversely, Black or Mixed-race women with lower education levels spent less on soft drinks, alcoholic beverages, and ready-to-eat foods compared to men, reflecting both economic constraints and distinct food practices ^21^. These patterns align with findings from other Brazilian research that has shown greater vulnerability to food and nutrition insecurity among Black women, regardless of income or education, indicating that overlapping social markers such as gender and race/skin color create additional structural barriers to adequate food access, even under more favorable socioeconomic conditions ^27^.

Another notable finding from the included studies was the higher food expenditure observed in households headed by Black or Mixed-race individuals, suggesting a potential additional financial burden faced by these groups in accessing food ^28^. Data from the POF 2017-2018 show that 44.4% of Brazilians living in families with a Black or Mixed-race reference person struggle to pay monthly expenses, including food ^29^. These results reveal structural inequalities embedded in food environments that directly affect the ability to choose and consume healthy foods.

Based on the limited and emerging evidence, this review suggests that gender and race/skin color may shape access to food. The concept of gender here refers to the social, historical, and cultural constructions associated with sexual differences, not merely biological characteristics, but also the roles, expectations, and power relations attributed to men, women, trans, non-binary, and other gender identities within a given society ^30^. In this sense, its impact on access to food, particularly healthy food, reflects an unequal social system that structurally privileges men and normalizes the subordination of women and other gender identities, sustained via social, cultural, political, and economic practices that reproduce gender inequalities ^31^. Brazil is among the countries with the highest gender inequality indexes ^32^, and women’s participation in the labor market, often involving double or triple work shifts combined with lower wages, affects their ability to access food ^33^ ^,^ ^34^. This situation is further aggravated for Black and Mixed-race women, who face both gender inequality and racism ^27^ ^,^ ^35^.

Racism, in turn, manifests as the differential treatment of minorities, promoting systematic inequalities in access to rights, opportunities, and resources ^36^. Beyond individual prejudice, racism must be understood as a structural element of Brazilian society. Structural racism is embedded within political, legal, economic, and social institutions, reproducing itself via seemingly neutral practices that, in effect, sustain the exclusion and marginalization of Black, Indigenous, and other racialized populations ^36^. It is a system that organizes social relations and upholds hierarchies without necessarily relying on individuals’ conscious intent ^36^.

Recognizing this structural dimension of racism is essential for understanding how racial inequalities are expressed in food access, acquisition, and availability in Brazil. These racial inequalities intersect with gender inequalities, so that Black women, for instance, experience a double vulnerability in accessing fundamental rights such as the human right to adequate food. This underscores the need for public food and nutrition security policies to simultaneously consider gender and race/skin color markers, acknowledging different levels of exclusion and prioritizing actions that promote equity. This includes territorial planning for healthy food supply in vulnerable areas and the design of programs that recognize the central role of women, particularly Black women, in household food management, ensuring their active participation in the design, implementation, and social oversight of public policies ^16^ ^,^ ^19^.

Regarding public policy implications, the studies included in this review showed recommendations aimed at policymakers. Most of these recommendations referred to general policies applicable across contexts, but few considered different levels of vulnerability and inequities in access. Among them, the implementation of public food and nutrition security facilities, promotion of urban agriculture, reduction of healthy food prices, and regulatory measures such as front-of-package nutrition labeling and food advertising regulation were most frequently cited ^16^ ^,^ ^17^ ^,^ ^18^ ^,^ ^19^ ^,^ ^21^. These are relevant actions that should be implemented nationwide to promote access to healthy foods. However, to ensure equity in access, such policies must account for intersecting forms of discrimination and inequality. Accordingly, researchers recommend prioritizing territories with the greatest need for food access policies and actively combating all forms of racial and gender discrimination as an essential condition for building healthier and fairer food environments ^16^ ^,^ ^17^ ^,^ ^18^ ^,^ ^19^ ^,^ ^21^.

Beyond the recommendations identified in the included studies, this review proposes a set of actions directly informed by the main findings and gaps mapped in the literature. These proposals respond to the concentration of food access barriers in socially and racially marginalized territories, the predominance of studies focused on food outlet availability, and the limited incorporation of gender and race/skin color intersectionality in existing analyses. Focusing on promoting gender and racial equity in food access, the proposed actions align with ongoing public policies in Brazil and reinforce the need to integrate food and nutrition security initiatives with an explicit intersectional approach. The proposals are systematized in Box 5.

**Box 5:** Proposals for incorporating racial and gender equity into public food and nutrition security policies.

AXIS	PROPOSITION
Peripheral territories	Map and prioritize territories with a high concentration of racial minorities, such as urban peripheries, *quilombola* communities, rural settlements, and agrarian reform areas, for food and nutrition security actions
Urban planning	Integrate the fight against food deserts and food swamps into participatory urban planning, mapping, and prioritizing territories within food and nutrition security policies
Social participation	Ensure the active participation of Black women and community leaders in food and nutrition security councils and forums, as well as in the design, implementation, and monitoring of public policies
Subsidies and incentives	Create incentive and/or subsidy policies for the acquisition of unprocessed and minimally processed foods targeted at households headed by Black women in situations of vulnerability
Food and nutrition education	Develop food and nutrition education campaigns and programs with anti-racist and feminist approaches, prioritizing the most vulnerable groups and valuing traditional food cultures, popular knowledge, and regional foods
Intersectoral monitoring	Incorporate gender, race/skin color, and socioeconomic status indicators into the monitoring and evaluation systems of food and nutrition security policies
Strengthening local production and trade	Provide financial support to Black women farmers and food entrepreneurs engaged in short and local food supply chains, promoting and subsidizing the availability of unprocessed and minimally processed foods, as well as dishes prepared from these foods

Some of the proposed actions already resonate with concrete public policy initiatives underway in Brazil. One example is the Feed Cities Strategy (*Estratégia Alimenta Cidades*), developed by the Brazilian Ministry of Social Development and Assistance, Family, and the Fight Against Hunger, which mapped food deserts in Brazilian municipalities and prioritized vulnerable territories in the formulation of local food and nutrition security strategies through situational diagnostics. This initiative can serve as a reference for both urban planning and territorial prioritization, as it integrates territorial diagnostics into the implementation of actions to expand access to healthy foods. The central challenge, however, lies in translating these diagnostics into effective territorial interventions. Regarding social participation, initiatives such as the Municipal Councils for Food and Nutrition Security (COMSEA, acronym in Portuguese) represent key spaces to ensure the active involvement of communities and local leaders, which can be strengthened by the deliberate inclusion of Black women and representatives of traditional territories.

In the domain of strengthening local production, programs such as the Brazilian Food Acquisition Program (PAA, acronym in Portuguese) and the Brazilian National School Feeding Program (PNAE, acronym in Portuguese) stand out for their potential to directly benefit Black women farmers and food entrepreneurs, particularly when linked to technical assistance, rural credit, and targeted public procurement. In the field of food and nutrition education, several civil society organizations, such as the Alliance for Adequate and Healthy Food, have developed educational materials and campaigns that value traditional food knowledge, strengthening the connection between culture, identity, and food. These examples demonstrate that it is possible to advance public policies that incorporate racial and gender equity as structuring principles, provided they are grounded in a deep understanding of the inequalities shaping the Brazilian food environment.

Furthermore, recommendations from the High Level Panel of Experts on Food Security and Nutrition (HLPE) and the Pan American Health Organization (PAHO) reinforce the need for universal regulatory measures, such as taxation of ultra-processed foods, regulation of their advertising, and front-of-package nutrition labeling ^37^ ^,^ ^38^ ^,^ ^39^ ^,^ ^40^ ^,^ ^41^ ^,^ ^42^, combined with targeted actions for peripheral and racialized communities. This dual strategy is indispensable for advancing equity.

As a finding of this scoping review, the recurring limitations reported by the included studies highlight structural gaps in the current literature. The main limitation identified by the authors relates to the use of secondary data, particularly on food outlets, which may hinder a more comprehensive understanding of food environments in vulnerable regions, especially due to the exclusion of informal and alternative food markets. However, this is a common limitation in studies of food environments in Brazil, given the country’s large territory ^43^. In such cases, one option to improve data quality is to conduct data validation, which can be performed virtually using tools such as street-level visualization ^44^ ^,^ ^45^ ^,^ ^46^.

This scoping review also identified gaps in the literature, such as the absence of studies specifically addressing the North and Central-West regions and limited research in the Northeast, which is significant given the cultural diversity across Brazilian regions and states. A predominance of studies based on secondary data and ecological designs was also observed. The small number of Brazilian studies on food access, acquisition, and availability that disaggregate data by gender and race/skin color underscores the need for expanded research in this area to expose existing inequities and advocate for public policies that are sensitive to the structural inequalities affecting historically marginalized groups. Additionally, the lack of intersectional approaches limits a broader understanding of the phenomena analyzed.

A particular gap lies in the scarcity of investigations explicitly incorporating gender as an analytical marker, which restricts comprehension of the different ways in which women, men, and other identities experience food access. This gap is noteworthy given the availability of national databases such as the POF, which have the potential to support more detailed analyses of food inequalities but remain underutilized in this field.

This review has its own limitations, which should be acknowledged. The analysis did not capture the diversity of gender identities beyond the cisgender and transgender binary, limiting the recognition of gender minorities. Moreover, as expected for scoping reviews, no assessment of methodological quality was conducted, and the review protocol was not prospectively registered, which may have reduced transparency, although protocol registration is not required under PRISMA-ScR guidelines. Finally, the same search string was applied across PubMed, Scopus, and Web of Science, which ensured consistency but did not account for database-specific syntax or indexing differences.

Taken together, the small number of included studies, their methodological profile, and the limited incorporation of intersectional analyses restrict any attempt to generalize or quantify inequalities. Accordingly, the primary contribution of this exploratory scoping review lies in mapping the existing evidence and, above all, in identifying critical gaps in the literature, rather than providing definitive estimates of gender- and race-based food inequalities.

## Final considerations

This scoping review enabled mapping evidence on food access, acquisition, and availability in Brazil, considering intersections of gender and race/skin color. The findings reveal structural inequalities in food access, with more pronounced barriers for racialized populations, particularly women, who face greater challenges in accessing unprocessed and minimally processed foods. The analysis showed a concentration of food deserts and food swamps in territories with higher social vulnerability, as well as distinct patterns of food acquisition and expenditure according to gender and race/skin color. However, there is a scarcity of studies that simultaneously address both gender and race/skin color markers, highlighting a significant gap in national scientific production.

The recommendations extracted from the analyzed studies underscore the importance of public policies that not only expand access to healthy foods but also confront the social and structural inequalities that shape food environments. The findings, therefore, contribute to informing the formulation of more fair and effective public policies that guarantee the human right to adequate food for the entire Brazilian population in an equitable manner.

## Data Availability

The sources of information used in the study are indicated in the body of the article.

## Material Suplementar

Material Suplementar
