# Supplementary material for: Inequalities in access to food in Brazil: a scoping review with a racial and gender focus and policy recommendations
Source: Cad Saude Publica. 2026 Jul 6;42:e00211325. doi: 10.1590/0102-311XEN211325 (PMC13335779; doi:10.1590/0102-311XEN211325)
Supplement: Material Suplementar [file 1678-4464-csp-42-EN211325-s.pdf]

SUPPLEMENTARY MATERIAL

Search strategy for the study “Inequalities in access to food in Brazil: a scoping review with a racial and gender focus and policy recommendations.”

Database	Search Strategy
PubMed, Web of Science and Scopus	((“Brasil” OR “Brazil”) AND (("Access to Food" OR “Food Access” OR “Access, Food” OR "Availability of Food" OR "Food Availability" OR "Availability, Food" OR “Food Acquisition” OR “Acquisition, Food” OR “Acquisition of Food” OR “Food Purchase” OR “Purchase, Food” OR “Purchase of Food” OR “Food Affordability” OR “Affordability of Food” OR “Affordability, Food”) OR ("Food"[Mesh] OR "Foods")) AND (("Racial Groups"[Mesh] OR "Group, Racial" OR "Groups, Racial" OR "Racial Group" OR "Race" OR "Races" OR "Racial Stocks" OR "Racial Stock" OR "Stock, Racial" OR "Stocks, Racial") OR ("Race Factors"[Mesh] OR "Race Factor" OR "Racial Factors" OR "Racial Factor") OR ("Black People"[Mesh] OR "Black Peoples" OR "People, Black" OR "Black Person" OR "Black Persons" OR "Person, Black" OR "Persons, Black" OR "Negroid Race" OR "Negroid Races" OR "Race, Negroid" OR "African Continental Ancestry Group") OR (“Ethnicity” OR "Ethnic group" OR "Ethnic-racial group" OR "Skin color" OR “Skin colour” OR "Ethnic minorities" OR "Racial minorities" OR "Ethnic-racial minorities" OR "Ethnic disparities" OR "Ethnic inequalities" OR "Racial disparities" OR "Racial inequalities" OR "Ethnic-racial disparities" OR "Ethnic-racial inequalities" OR "Racial discrimination" OR “Racism” OR "Structural Racism") OR ("Gender" OR “Sex” OR (“Woman” OR “Women”) OR (“Men” OR “Man”)) OR ("Social Vulnerability"[Mesh] OR "Social Vulnerabilities" OR "Vulnerabilities, Social" OR "Vulnerability, Social") OR (“Iniquity” OR “Iniquities” OR “Social Iniquity” OR “Social Iniquities”)))
SciELO	(((Brasil) OR (Brazil)) AND ((Access to Food) OR (Food Access) OR (Access, Food) OR (Availability of Food) OR (Food Availability) OR (Availability, Food) OR (Food Acquisition) OR (Acquisition, Food) OR (Acquisition of Food) OR (Food Purchase) OR (Purchase, Food) OR (Purchase of Food) OR (Food Affordability) OR (Affordability of Food) OR (Affordability, Food) OR (Foods) OR (Food)) AND ((Racial Groups) OR (Group, Racial) OR (Groups, Racial) OR (Racial Group) OR (Race) OR (Races) OR (Racial Stocks) OR (Racial Stock) OR (Stock, Racial) OR (Stocks, Racial) OR (Race Factors) OR (Race Factor) OR (Racial Factors) OR (Racial Factor) OR (Black People) OR (Black Peoples) OR (People, Black) OR (Black Person) OR (Black Persons) OR (Person, Black) OR (Persons, Black) OR (Negroid Race) OR (Negroid Races) OR (Race, Negroid) OR (African Continental Ancestry Group) OR (Ethnicity) OR (Ethnic group) OR (Ethnic-racial group) OR (Skin color) OR (Skin colour) OR (Ethnic minorities) OR (Racial minorities) OR (Ethnic-racial minorities) OR (Ethnic disparities) OR (Ethnic inequalities) OR (Racial disparities) OR (Racial inequalities) OR (Ethnic-racial disparities) OR (Ethnic-racial inequalities) OR (Racial discrimination) OR (Racism) OR (Structural Racism) OR (Gender) OR (Sex) OR (Woman) OR (Women) OR (Man) OR (Men) OR (Social Vulnerability) OR (Social Vulnerabilities) OR (Vulnerabilities, Social) OR (Vulnerability, Social) OR (Iniquity) OR (Iniquities) OR (Social Iniquity) OR (Social Iniquities)))
